# Culture‐Confirmed Significant Bacteriuria and Antimicrobial Susceptibility Patterns Among Pregnant Women Attending Antenatal Care at a District Hospital in Ghana

**DOI:** 10.1155/bmri/7197672

**Published:** 2026-11-14

**Authors:** Mina Ofosu, Vuur Edward Domar, Patricia Sam Barko

**Affiliations:** ^1^ Department of Laboratory Technology Faculty of Health Sciences Kumasi Technical University Kumasi Ghana kstu.edu.gh

**Keywords:** antibacterial agents, bacterial, bacteriuria, drug resistance, Ghana, microbial sensitivity tests, pregnant women, urinary tract infections

## Abstract

**Background:**

Urinary tract infections are common during pregnancy and may be associated with adverse maternal and neonatal outcomes. Antimicrobial resistance can complicate treatment and varies across healthcare settings. This study assessed the prevalence of culture‐confirmed significant bacteriuria and antimicrobial susceptibility patterns among pregnant women attending antenatal care at Father Thomas Rooney Catholic Hospital, Ghana.

**Methods:**

A cross‐sectional study was conducted among 200 pregnant women. Clean‐catch midstream urine samples were cultured using standard microbiological procedures, with significant bacteriuria defined as ≥ 10^5^ CFU/mL of a single organism. Isolates were identified using conventional biochemical methods, and susceptibility was determined by Kirby–Bauer disk diffusion according to CLSI 2024 guidelines. Associations with culture positivity were assessed using chi‐square or Fisher′s exact tests, followed by binary logistic regression restricted to two predictors. Adjusted odds ratios (AORs) with 95% confidence intervals (CIs) were reported.

**Results:**

Culture‐confirmed significant bacteriuria was identified in 23/200 participants (11.5%). Six species were isolated, with *Escherichia coli* predominant (8/23, 34.8%), followed by *Staphylococcus aureus* (5/23, 21.7%), *Klebsiella pneumoniae* (4/23, 17.4%), *Proteus mirabilis* (3/23, 13.0%), *Pseudomonas aeruginosa* (2/23, 8.7%), and *Proteus vulgaris* (1/23, 4.3%). Two *P. aeruginosa* isolates were excluded from susceptibility analysis, leaving 21 isolates. Among 16 gram‐negative isolates, susceptibility to amikacin and gentamicin was 81.3% each, ampicillin susceptibility was 31.3% with resistance in 43.8%, and ceftriaxone resistance was 62.5%. Tight underwear use (AOR = 6.56; 95% CI: 2.36–18.22; *p* < 0.001) and douching or deodorant use (AOR = 8.50; 95% CI: 2.83–25.54; *p* < 0.001) were associated with culture positivity.

**Conclusion:**

Significant bacteriuria was common in this antenatal population, with retained aminoglycoside activity but low ampicillin susceptibility and substantial ceftriaxone resistance. These findings support considering urine culture at the first antenatal visit where laboratory capacity permits, establishing facility‐level antibiograms, and including genital hygiene counseling in antenatal education. Larger multicenter studies are needed to confirm these findings.

## 1. Introduction

Urinary tract infections (UTIs) are among the most common bacterial infections during pregnancy and are associated with adverse maternal and perinatal outcomes, including pyelonephritis, preterm birth, and low birth weight when inadequately managed [1, 2]. In pregnancy, urinary infection may present as asymptomatic bacteriuria (ASB), cystitis, or pyelonephritis. ASB is significant bacteriuria without urinary symptoms and is clinically important because untreated cases can progress to symptomatic infection, including pyelonephritis [3]. The burden of urinary infection in pregnancy varies across populations and geographical settings. A global meta‐analysis estimated a pooled UTI prevalence in pregnancy of 13.2% (95% confidence interval [CI]: 12.8–13.6), with the highest burden in Africa [4]. An African meta‐analysis estimated the pooled prevalence of ASB among pregnant women at 11.1% (95% CI: 7.8–14.4) [5]. Physiological changes in pregnancy, including ureteral dilatation, reduced bladder tone, urinary stasis, and altered urinary composition, increase susceptibility to bacteriuria [6]. Behavioral and clinical factors such as genital hygiene practices, vaginal douching, and previous UTI have been associated with bacteriuria in observational studies, although such associations do not establish causality [7]. *Escherichia coli* is consistently reported as the predominant uropathogen in pregnancy, although species distribution varies across populations and healthcare settings [8]. Antimicrobial resistance among uropathogens is an increasing concern, and studies from Ghana and other African settings have reported substantial resistance to ampicillin, cotrimoxazole, and ceftriaxone, whereas susceptibility to selected aminoglycosides has been retained in some settings [9]. Evidence from Ghana demonstrates geographical variation in the prevalence of bacteriuria and the antimicrobial susceptibility of urinary isolates. Among first‐time antenatal attendees at a secondary health facility in Ghana, bacterial culture positivity was reported in 21.5% of participants, and resistance among the isolated bacteria ranged from 50% to 100% across the antibiotics tested [10]. Studies among pregnant women in Bolgatanga and Ho reported ASB prevalences of 17.5% and 13.7%, respectively, with resistance to several commonly tested antibiotics [11, 12]. In the Ho study, all recovered isolates were resistant to amoxicillin–clavulanate, although 87% were susceptible to gentamicin [12]. Beyond antenatal populations, a hospital‐based study in northern Ghana reported 100% ampicillin resistance among gram‐negative isolates and multidrug resistance in 92% of the recovered uropathogens [13]. A retrospective study in rural Kpando also documented substantial antimicrobial resistance among urinary isolates, with a mean resistance to 8.6 of the 11 antibiotics tested per patient [14]. Although these findings arise from different study populations and settings and should not be interpreted as nationally representative estimates, they demonstrate the importance of local susceptibility surveillance. However, published data on culture‐confirmed significant bacteriuria and antimicrobial susceptibility among pregnant women attending district‐level facilities in the Western Region remain limited. Facility‐level evidence from Father Thomas Rooney Catholic Hospital (FTRCH) may therefore help characterize locally encountered urinary isolates and their susceptibility profiles.

## 2. Materials and Methods

### 2.1. Study Area

The study was conducted at the bacteriology unit of FTRCH, located in the Wassa Amenfi West Municipal District of the Western Region of Ghana (Figure 1). The facility serves as a referral and antenatal care center for women from the municipality and surrounding communities and provides both outpatient and inpatient services. The facility also serves clients enrolled under the National Health Insurance Scheme (NHIS) and receives a substantial number of patients from the surrounding population.

**Figure 1:**
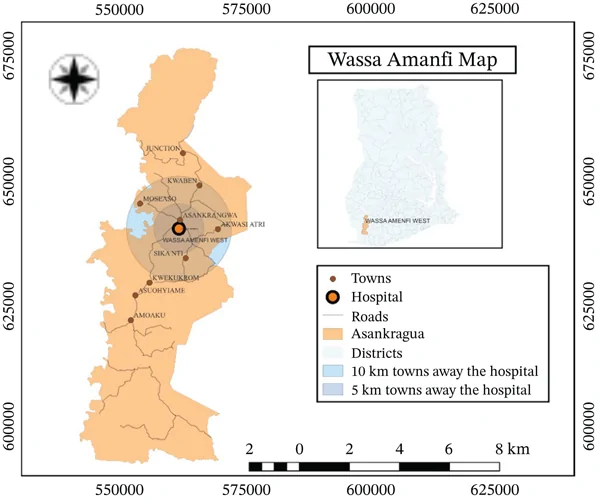
Map of Wassa Amenfi West Municipal showing major towns, with the study site (Father Thomas Rooney Catholic Hospital) highlighted at Asankragwa. *Source:* Ghana Statistical Service & Survey and Mapping Division (2019).

### 2.2. Study Design and Study Period

A hospital‐based cross‐sectional study was conducted from May to July 2025 among pregnant women attending antenatal care at the study facility. Pregnant women were recruited irrespective of the presence or absence of urinary symptoms. Participants provided clean‐catch midstream urine specimens and completed a structured questionnaire capturing relevant sociodemographic, obstetric, behavioral, and clinical information. A convenience sampling approach was used because recruitment was based on the availability of eligible participants during the study period and the practical feasibility of specimen collection and laboratory processing.

### 2.3. Study Population and Eligibility Criteria

The study population comprised pregnant women attending antenatal care at the study facility during the study period. Pregnant women of all ages, parity groups, gestational ages, and socioeconomic backgrounds were eligible to participate. Women who were receiving antibiotic therapy at the time of recruitment were excluded because recent antimicrobial exposure could affect urine culture results and antimicrobial susceptibility patterns.

### 2.4. Sample Size Determination

The minimum sample size was estimated using the Cochran formula:

n=Z2.p1−pe2,

where *n* is the required sample size, *Z* is the standard normal deviate corresponding to a 95% confidence level (1.96), *p* is the estimated prevalence, and *e* is the desired margin of error. In the absence of sufficiently specific local prevalence data for the study population, a prevalence of 50% was assumed to provide the maximum sample size, with a 7% margin of error. This yielded a minimum sample size of 196 participants. The target sample size was increased to 200 participants to provide a small allowance for potential incomplete questionnaires or missing data and to facilitate practical field implementation.

### 2.5. Study Outcome Definition

The primary outcome was culture‐confirmed significant bacteriuria, defined as the isolation of a single bacterial organism from a clean‐catch midstream urine specimen at a concentration of ≥ 10^5^ colony‐forming units (CFUs)/mL. Participants were enrolled irrespective of urinary symptoms; therefore, the outcome included both asymptomatic and symptomatic women. This outcome is distinct from a clinical diagnosis of symptomatic UTI.

### 2.6. Urine Sample Collection, Transport, and Processing

Participants were instructed on the collection of clean‐catch midstream urine specimens. Each participant provided a urine specimen into a sterile, leak‐proof, appropriately labeled universal specimen container. Specimens were transported to the bacteriology laboratory within 1 hour of collection at ambient temperature. Where immediate processing was not possible, specimens were refrigerated at 4°C and processed within 4 hours of collection. Each specimen was mixed gently before inoculation. Using a calibrated 10‐*μ*L inoculating loop, urine was inoculated onto cysteine lactose electrolyte‐deficient (CLED) agar. Plates were incubated aerobically at 37°C for 18–24 hours. A specimen yielding a single bacterial organism at a concentration of ≥ 10^5^ CFU/mL was classified as having significant bacteriuria. Specimens with mixed growth involving three or more organisms or heavy mixed skin flora without a predominant organism were classified as contaminated and were not considered positive for significant bacteriuria.

### 2.7. Bacterial Identification

Bacterial isolates were identified using colony morphology, Gram staining, and conventional biochemical tests, including catalase, coagulase, citrate utilization, indole production, oxidase, motility, and urease tests, as appropriate for the suspected organism. *E. coli* was identified as a gram‐negative lactose‐fermenting organism that was indole‐positive, citrate‐negative, and oxidase‐negative. *Klebsiella pneumoniae* was identified as a lactose‐fermenting, nonmotile, indole‐negative, citrate‐positive, and urease‐positive organism. *Pseudomonas aeruginosa* was identified based on its characteristic nonlactose‐fermenting colony morphology, oxidase positivity, motility, growth at 42°C, and pigment production. *Proteus* species were identified by their characteristic swarming growth, motility, and urease positivity. *Proteus mirabilis* and *Proteus vulgaris* were differentiated using indole production, with *P. mirabilis* being indole negative and *P. vulgaris* indole positive. *Staphylococcus aureus* was identified as a gram‐positive coccus occurring in clusters and showing catalase and coagulase positivity.

### 2.8. Antimicrobial Susceptibility Testing (AST)

AST was performed using the Kirby–Bauer disk diffusion method on Mueller–Hinton agar. Bacterial suspensions were prepared to approximately 0.5 McFarland turbidity, evenly inoculated onto Mueller–Hinton agar, and antimicrobial disks were applied to the inoculated surface. Plates were incubated at 37°C for 18–24 hours, after which inhibition zones were measured in millimeters. For gram‐negative isolates, the antimicrobial disks tested were ampicillin (10 *μ*g), gentamicin (10 *μ*g), ciprofloxacin (5 *μ*g), ceftriaxone (30 *μ*g), cefotaxime (30 *μ*g), chloramphenicol (30 *μ*g), amikacin (30 *μ*g), meropenem (10 *μ*g), trimethoprim–sulfamethoxazole/cotrimoxazole (25 *μ*g), tetracycline (30 *μ*g), and cefuroxime (30 *μ*g). Vancomycin (30 *μ*g) was tested against gram‐positive isolates. Interpretation of susceptibility results was based on the Clinical and Laboratory Standards Institute (CLSI) M100, 34th edition [15], using organism‐specific interpretive criteria where applicable. The antimicrobial panel reflected the drugs available for routine testing at the study laboratory and commonly used antimicrobial agents for bacterial infections in the study setting. Nitrofurantoin and amoxicillin–clavulanic acid were not included because the corresponding antimicrobial disks were unavailable during the study period.

### 2.9. Quality Control

Quality control procedures were implemented during culture and AST. Culture media, including CLED and Mueller–Hinton agar, were prepared and subjected to appropriate quality‐control procedures before use. Reference strains *E. coli* ATCC 25922, *P. aeruginosa* ATCC 27853, and *S. aureus* ATCC 25923 were used for quality control of bacterial identification and AST, as applicable. Susceptibility testing was considered acceptable when inhibition zones fell within the relevant CLSI quality‐control ranges. Questionable laboratory findings were repeated once where necessary.

### 2.10. Questionnaire Development and Data Collection

A structured questionnaire was developed by the investigators after reviewing comparable studies conducted among pregnant women in Ghana and related settings. The questionnaire was reviewed by two senior laboratory scientists and one obstetric clinician for content validity. It was then pretested among 10 pregnant women attending antenatal care at a comparable facility outside the study site. These participants were not included in the final study sample, and wording was modified where necessary following the pretest. Data collectors received 2 days of training covering informed consent procedures, questionnaire administration, specimen identification and labeling, and appropriate handling of study materials. Information collected included relevant sociodemographic, obstetric, behavioral, and clinical characteristics. For the behavioral variable concerning underwear use, tight underwear was defined according to the participant′s reported routine use of underwear perceived to fit tightly around the genital region. A history of douching or deodorant use was recorded as a binary variable (any vs. none) based on participant‐reported use of any intravaginal product, including vinegar or baking soda solutions, antiseptics such as povidone‐iodine, and scented products. Behavioral variables were analyzed as binary exposures (any vs. none); information on the specific products used was collected but not analyzed separately. The final questionnaire used for data collection is provided as File S1.

### 2.11. Statistical Analysis

Data were entered and initially organized in Microsoft Excel 2019, then analyzed in IBM SPSS Statistics Version 26 and RStudio. Descriptive statistics were used to summarize participant characteristics, culture results, bacterial isolates, and antimicrobial susceptibility patterns. Categorical variables were presented as frequencies and percentages. Associations between participant characteristics and culture‐confirmed significant bacteriuria were first assessed using the Pearson chi‐square test or Fisher′s exact test, as appropriate, depending on expected cell counts. Variables with a univariable *p* value < 0.25 were considered for inclusion in multivariable analysis. Given that only 23 participants had culture‐confirmed significant bacteriuria, the multivariable binary logistic regression model was restricted to two predictors, namely tight underwear use and douching or deodorant use, to maintain an events‐per‐variable ratio above 10 and reduce the risk of overfitting. Age group and gestational age were not retained in the final model because they did not meet the *p* < 0.25 screening threshold and contained categories with zero culture‐positive participants. Crude odds ratios (CORs) and adjusted odds ratios (AORs) with 95% CIs were reported. Statistical significance was set at *p* < 0.05. Model fit was assessed using the Hosmer–Lemeshow goodness‐of‐fit test, and multicollinearity among predictors was assessed using variance inflation factors (VIFs). Given the small number of culture‐positive events, adjusted estimates were interpreted as associations rather than definitive independent risk factors.

### 2.12. Ethics Approval and Consent to Participate

Ethical clearance for this study was obtained from the Institute of Research, Innovation and Development (IRID) Ethics Committee, Kumasi, Ashanti Region, Ghana (Reference: IRID/EC2025/HS0011). Written informed consent was obtained from all participants before enrolment in the study. All procedures involving human participants were performed in accordance with the ethical standards of the institutional research committee and with the 1964 Declaration of Helsinki and its later amendments, or comparable ethical standards. Participants with culture‐confirmed significant bacteriuria were referred to the clinical team for further assessment and management, and their laboratory reports were provided to facilitate this process.

## 3. Results

### 3.1. Prevalence of Culture‐Confirmed Significant Bacteriuria

Of the 200 pregnant women enrolled, 23 (11.5%) had culture‐confirmed significant bacteriuria (≥ 10^5^ CFU/mL of a single organism), and 177 (88.5%) had negative cultures (Figure 2).

**Figure 2:**
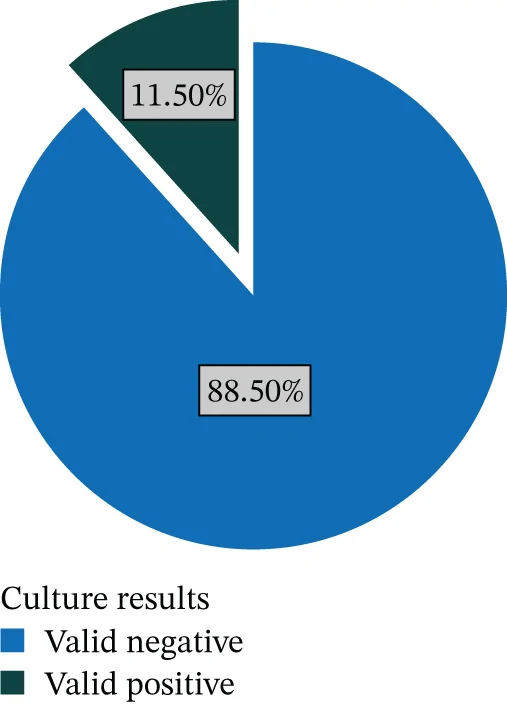
Prevalence of culture‐confirmed significant bacteriuria among pregnant women (*n* = 200).

### 3.2. Bacterial Isolate and Distribution

A total of 23 bacterial isolates representing six species were recovered from the 23 culture‐positive urine specimens. *E. coli* was the most frequently isolated organism, accounting for 8/23 (34.8%) isolates, followed by *S. aureus* 5/23 (21.7%), *K. pneumoniae* 4/23 (17.4%), *P. mirabilis* 3/23 (13.0%), *P. aeruginosa* 2/23 (8.7%), and *P. vulgaris* 1/23 (4.3%) (Figure 3). Species‐specific findings for organisms represented by one or two isolates should be interpreted cautiously because of the small denominators.

**Figure 3:**
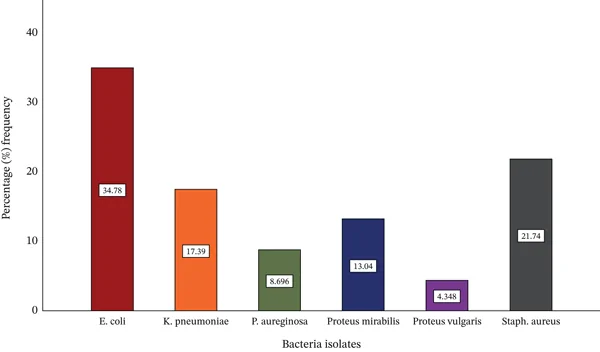
Distribution and percentage frequency of bacterial isolates recovered from urine specimens (*n* = 23).

### 3.3. Antibiotic Susceptibility Patterns

Of the 23 bacterial isolates recovered, 21 were included in the primary antimicrobial susceptibility analysis. The two *P. aeruginosa* isolates were excluded because their observed susceptibility profiles were considered unreliable for interpretation. The 21 isolates included eight *E. coli*, four *K. pneumoniae*, three *P. mirabilis*, one *P. vulgaris*, and five *S. aureus*.

#### 3.3.1. Antimicrobial Susceptibility of Gram‐Negative Isolates

Sixteen gram‐negative isolates were included in the primary antimicrobial susceptibility analysis. Amikacin and gentamicin demonstrated the highest pooled susceptibility, with 13/16 (81.3%) isolates susceptible to each antimicrobial. This was followed by cotrimoxazole, with 12/16 (75.0%) susceptible isolates. Ciprofloxacin and tetracycline each showed 11/16 (68.8%) susceptibility, whereas meropenem and cefuroxime each showed 10/16 (62.5%) susceptibility. Lower pooled susceptibility was observed for chloramphenicol and cefotaxime, each with 6/16 (37.5%) susceptible isolates, and for ampicillin and ceftriaxone, each with 5/16 (31.3%) susceptible isolates. Resistance to ceftriaxone was observed in 10/16 (62.5%) gram‐negative isolates, whereas resistance to ampicillin occurred in 7/16 (43.8%) isolates. Species‐specific distributions are presented in Table 1.

**Table 1:** Antimicrobial susceptibility patterns of gram‐negative urinary isolates (*n* = 16).

Antibiotic	Pattern	*E. coli* (*n* = 8)	*K. pneumoniae* (*n* = 4)	*P. mirabilis* (*n* = 3)	*P. vulgaris* (*n* = 1)	Pooled gram‐negative (*n* = 16)
Ampicillin (AMP)	S	1 (12.5)	2 (50.0)	2 (66.7)	0 (0.0)	5 (31.3)
	I	2 (25.0)	1 (25.0)	0 (0.0)	1 (100.0)	4 (25.0)
	R	5 (62.5)	1 (25.0)	1 (33.3)	0 (0.0)	7 (43.8)

Gentamicin (GEN)	S	6 (75.0)	3 (75.0)	3 (100.0)	1 (100.0)	13 (81.3)
	I	1 (12.5)	0 (0.0)	0 (0.0)	0 (0.0)	1 (6.3)
	R	1 (12.5)	1 (25.0)	0 (0.0)	0 (0.0)	2 (12.5)

Amikacin (AMK)	S	8 (100.0)	2 (50.0)	3 (100.0)	0 (0.0)	13 (81.3)
	I	0 (0.0)	0 (0.0)	0 (0.0)	1 (100.0)	1 (6.3)
	R	0 (0.0)	2 (50.0)	0 (0.0)	0 (0.0)	2 (12.5)

Ciprofloxacin (CIP)	S	7 (87.5)	3 (75.0)	0 (0.0)	1 (100.0)	11 (68.8)
	I	0 (0.0)	0 (0.0)	0 (0.0)	0 (0.0)	0 (0.0)
	R	1 (12.5)	1 (25.0)	3 (100.0)	0 (0.0)	5 (31.3)

Chloramphenicol (CHL)	S	2 (25.0)	3 (75.0)	1 (33.3)	0 (0.0)	6 (37.5)
	I	1 (12.5)	1 (25.0)	0 (0.0)	0 (0.0)	2 (12.5)
	R	5 (62.5)	0 (0.0)	2 (66.7)	1 (100.0)	8 (50.0)

Cefotaxime (CTX)	S	3 (37.5)	2 (50.0)	1 (33.3)	0 (0.0)	6 (37.5)
	I	0 (0.0)	1 (25.0)	0 (0.0)	0 (0.0)	1 (6.3)
	R	5 (62.5)	1 (25.0)	2 (66.7)	1 (100.0)	9 (56.3)

Ceftriaxone (CTR)	S	3 (37.5)	0 (0.0)	1 (33.3)	1 (100.0)	5 (31.3)
	I	1 (12.5)	0 (0.0)	0 (0.0)	0 (0.0)	1 (6.3)
	R	4 (50.0)	4 (100.0)	2 (66.7)	0 (0.0)	10 (62.5)

Meropenem (MEM)	S	5 (62.5)	2 (50.0)	3 (100.0)	0 (0.0)	10 (62.5)
	I	1 (12.5)	1 (25.0)	0 (0.0)	0 (0.0)	2 (12.5)
	R	2 (25.0)	1 (25.0)	0 (0.0)	1 (100.0)	4 (25.0)

Tetracycline (TET)	S	6 (75.0)	2 (50.0)	2 (66.7)	1 (100.0)	11 (68.8)
	I	1 (12.5)	2 (50.0)	0 (0.0)	0 (0.0)	3 (18.8)
	R	1 (12.5)	0 (0.0)	1 (33.3)	0 (0.0)	2 (12.5)

Cefuroxime (CRX)	S	6 (75.0)	2 (50.0)	2 (66.7)	0 (0.0)	10 (62.5)
	I	0 (0.0)	0 (0.0)	0 (0.0)	1 (100.0)	1 (6.3)
	R	2 (25.0)	2 (50.0)	1 (33.3)	0 (0.0)	5 (31.3)

Cotrimoxazole (COT)	S	5 (62.5)	4 (100.0)	2 (66.7)	1 (100.0)	12 (75.0)
	I	1 (12.5)	0 (0.0)	0 (0.0)	0 (0.0)	1 (6.3)
	R	2 (25.0)	0 (0.0)	1 (33.3)	0 (0.0)	3 (18.8)

*Note:* Values are *n* (%). Breakpoints per CLSI M100, 34th ed. (2024).

Abbreviations: AMK, amikacin; AMP, ampicillin; CHL, chloramphenicol; CIP, ciprofloxacin; COT, cotrimoxazole (trimethoprim–sulfamethoxazole); CRX, cefuroxime; CTR, ceftriaxone; CTX, cefotaxime; GEN, gentamicin; I, intermediate; MEM, meropenem; R, resistant; S, susceptible; TET, tetracycline.

#### 3.3.2. Antimicrobial Susceptibility of *S. aureus*

Five *S. aureus* isolates were included in the antimicrobial susceptibility analysis. Cotrimoxazole showed the highest activity, with all five isolates susceptible (5/5, 100.0%). Amikacin showed susceptibility in 4/5 (80.0%) isolates, whereas gentamicin, ciprofloxacin, and cefuroxime each showed susceptibility in 3/5 (60.0%) isolates. Resistance was highest for chloramphenicol, with 4/5 (80.0%) isolates resistant, followed by ampicillin and tetracycline, each with 3/5 (60.0%) resistant isolates. Vancomycin was tested by disk diffusion, but results were considered preliminary and excluded from the primary susceptibility interpretation. Full results are presented in Table 2.

**Table 2:** Antimicrobial susceptibility patterns of *Staphylococcus aureus* isolates (*n* = 5).

Antibiotic	Susceptible, *n* (%)	Intermediate, *n* (%)	Resistant, *n* (%)
Ampicillin	2 (40.0)	0 (0.0)	3 (60.0)
Gentamicin	3 (60.0)	0 (0.0)	2 (40.0)
Amikacin	4 (80.0)	0 (0.0)	1 (20.0)
Ciprofloxacin	3 (60.0)	0 (0.0)	2 (40.0)
Chloramphenicol	1 (20.0)	0 (0.0)	4 (80.0)
Cefotaxime	2 (40.0)	1 (20.0)	2 (40.0)
Ceftriaxone	3 (60.0)	1 (20.0)	1 (20.0)
Meropenem	2 (40.0)	1 (20.0)	2 (40.0)
Tetracycline	2 (40.0)	0 (0.0)	3 (60.0)
Cefuroxime	3 (60.0)	0 (0.0)	2 (40.0)
Cotrimoxazole	5 (100.0)	0 (0.0)	0 (0.0)
Vancomycin∗	4 (80.0)	0 (0.0)	1 (20.0)

*Note:* Asterisk “∗” denotes that vancomycin susceptibility was assessed by disk diffusion only and is reported as a preliminary screening finding; it was excluded from the primary susceptibility interpretation because definitive testing requires an MIC‐based method.

### 3.4. Exclusion of Two *P. aeruginosa* Isolates From Susceptibility Analysis

Two *P. aeruginosa* isolates were recovered from the culture‐positive specimens. Both isolates showed apparent susceptibility to ampicillin and cotrimoxazole, which is inconsistent with the intrinsic resistance profile of *P. aeruginosa*. Both isolates were retested once, and the anomalous results persisted. Identification was confirmed by nonlactose fermentation on CLED agar, oxidase positivity, motility, growth at 42°C, and characteristic pigment production. Because the susceptibility profiles could not be reconciled with the organism′s known intrinsic resistance, both isolates were excluded from the primary antimicrobial susceptibility analysis to avoid misleading interpretation. The primary susceptibility analysis therefore included 21 isolates.

### 3.5. Factors Associated With Culture‐Confirmed Significant Bacteriuria

The distribution of participants according to culture status and the corresponding crude and adjusted estimates of association are presented in Table 3. Percentages in Table 3 are row percentages within each category, calculated as the number of participants in that category divided by the total number of participants in the same category. Culture positivity was significantly associated with wearing tight underwear and a history of douching or deodorant use. Among participants who reported wearing tight underwear, 14/37 (37.8%) had culture‐confirmed significant bacteriuria, compared with 9/163 (5.5%) among those who did not (*p* < 0.001). Similarly, 18/58 (31.0%) participants who reported a history of douching or deodorant use had positive cultures, compared with 5/142 (3.5%) among those without such a history (*p* < 0.001). Age group and gestational age were not significantly associated with culture status (*p* = 0.328 and *p* = 0.428, respectively). Binary logistic regression was subsequently used to estimate the crude and adjusted associations. The multivariable model was restricted to two predictors, namely, wearing tight underwear and a history of douching or deodorant use, because age group and gestational age did not meet the *p* < 0.25 screening threshold and contained categories with zero culture‐positive participants. After adjustment for the other predictor in the model, participants who reported wearing tight underwear had higher odds of culture‐confirmed significant bacteriuria than those who did not (AOR = 6.56; 95% CI: 2.36–18.22; *p* < 0.001). A history of douching or deodorant use was also associated with higher adjusted odds of culture‐confirmed significant bacteriuria (AOR = 8.50; 95% CI: 2.83–25.54; *p* < 0.001). The final two‐predictor model showed adequate goodness‐of‐fit (Hosmer–Lemeshow *χ* ^2^ = 0.020, df = 2, *p* = 0.990), with no evidence of problematic multicollinearity (VIF = 1.093 for both predictors). Given the small number of culture‐positive events, these adjusted estimates should be interpreted as associations rather than definitive independent risk factors.

**Table 3:** Factors associated with culture‐confirmed significant bacteriuria among pregnant women (*N* = 200).

Variable	Category	Culture‐negative *n*/*N* (%)	Culture‐positive *n*/*N* (%)	COR (95% CI)	AOR (95% CI)	*p*
Age group (years)	15–20	15/18 (83.3)	3/18 (16.7)	Ref.	—	0.328^a^
	21–26	145/161 (90.1)	16/161 (9.9)	0.55 (0.14–2.11)	—	
	27–32	10/13 (76.9)	3/13 (23.1)	1.50 (0.25–8.98)	—	
	33–38	5/5 (100.0)	0/5 (0.0)	NE	—	
	> 38	2/3 (66.7)	1/3 (33.3)	2.50 (0.17–37.26)	—	

Gestational age (weeks)	1–12	106/119 (89.1)	13/119 (10.9)	Ref.	—	0.428^a^
	13–24	38/43 (88.4)	5/43 (11.6)	1.07 (0.36–3.21)	—	
	25–36	22/27 (81.5)	5/27 (18.5)	1.85 (0.60–5.73)	—	
	> 36	11/11 (100.0)	0/11 (0.0)	NE	—	

Wearing tight underwear	No	154/163 (94.5)	9/163 (5.5)	Ref.	Ref.	< 0.001^b^
	Yes	23/37 (62.2)	14/37 (37.8)	10.42 (4.05–26.80)	6.56 (2.36–18.22)	< 0.001^c^

Douching or deodorant use	No	137/142 (96.5)	5/142 (3.5)	Ref.	Ref.	< 0.001^b^
	Yes	40/58 (69.0)	18/58 (31.0)	12.33 (4.31–35.29)	8.50 (2.83–25.54)	< 0.001^c^

*Note:* Values are *n*/*N* (%) within each category (row percentages). The multivariable model was restricted to two predictors (tight underwear use; douching or deodorant use) to maintain an events‐per‐variable ratio above 10 given 23 events; age group and gestational age were excluded due to zero‐cell categories and failure to meet the *p* < 0.25 threshold. Estimates based on zero or one event are imprecise and should be interpreted descriptively.

Abbreviations: AOR, adjusted odds ratio; CI, confidence interval; COR, crude odds ratio; NE, not estimable (no culture‐positive participants); Ref., reference category.

^a^Pearson chi‐square or Fisher′s exact test, as appropriate.

^b^Univariable binary logistic regression (Wald test).

^c^Multivariable binary logistic regression (Wald).

## 4. Discussion

### 4.1. Prevalence of Culture‐Confirmed Significant Bacteriuria

This study identified culture‐confirmed significant bacteriuria in 23 of 200 pregnant women, giving a prevalence of 11.5%. Because participants were enrolled irrespective of urinary symptoms and the outcome was defined microbiologically at a threshold of ≥ 10^5^ CFU/mL, this estimate represents significant bacteriuria rather than clinically diagnosed UTI. The estimate is comparable with several Ghanaian reports, although substantial variation exists between studies. A study in Bolgatanga reported significant bacteriuria in 17.5% of pregnant women, whereas a study in Ho reported ASB in 13.7% [13, 14]. A study at Korle‐Bu Teaching Hospital reported an ASB prevalence of 11.5%, identical to the present estimate [16]. More recently, a study among first‐time antenatal attendees in Tamale reported bacterial culture positivity in 21.5% of participants [10]. The present estimate therefore falls within the range reported across Ghanaian antenatal populations rather than representing an unusually high or low finding. Methodological and population factors likely explain these differences. Studies limited to asymptomatic women or first‐time antenatal attendees often report higher prevalence than those including unselected populations, as selective recruitment concentrates women with distinct exposure profiles and healthcare‐seeking behaviors. Facility level also matters: Tertiary and referral hospitals tend to show higher prevalence than district facilities, plausibly due to referral of women with complications or prior treatment failure. Additional variation may reflect prior antimicrobial exposure, specimen collection practices, and the inclusion of symptomatic women in study populations. Comparable estimates have been reported elsewhere in Africa, including Nigeria and Ethiopia, though a systematic review highlighted wide variation across low‐resource settings due to differences in diagnostic thresholds, screening practices, and healthcare access [17]. A meta‐analysis of Ethiopian studies reported a pooled prevalence of 15.37% [18], higher than the present estimate, likely because clinical outcomes included symptomatic cases not meeting the ≥ 10^5^ CFU/mL threshold. In contrast, lower prevalence is typical in high‐income settings with established antenatal screening programs [19]. These contrasts underscore the need to interpret prevalence in relation to study population, sampling approach, clinical status, and microbiological definition. Given the prevalence of culture‐confirmed significant bacteriuria observed in this study, the findings may support consideration of urine culture at the first antenatal visit where laboratory capacity permits. Facility‐level susceptibility data may also help inform empirical treatment decisions, while larger multicenter studies are needed to provide more generalizable evidence.

### 4.2. Bacterial Isolates and Distribution

*E. coli* was the predominant isolate, accounting for 34.8% of culture‐positive specimens, consistent with reports from Bolgatanga and Korle‐Bu, where it comprised 42.9% and 43.6% of isolates, respectively [13, 16]. The lower proportion here may reflect the broader distribution of organisms within a small sample, differences in population characteristics, or prior antimicrobial exposure. Its predominance is biologically plausible, as intestinal strains can colonize the periurethral area, with uropathogenic variants possessing adhesins and other virulence factors that facilitate persistence [20]. Other isolates were broadly consistent with pregnancy‐associated urinary infections, though distributions varied. Likewise, a study in Ho reported *Pseudomonas* species as most frequent (26.1%), whereas *E. coli* accounted for only 21.7% [14]. Such variation may reflect ecological differences, healthcare exposure, specimen handling, antimicrobial use, or reliance on conventional biochemical identification [21]. The relatively high proportion of *S. aureus* (21.7%) warrants caution, as recovery from clean‐catch specimens does not confirm symptomatic infection. Because participants were enrolled irrespective of symptoms and ASB versus UTI was not classified, these findings represent microbiological recovery rather than definitive attribution of infection, a caution especially relevant for atypical urinary organisms. These findings highlight the need to strengthen clean‐catch collection technique through patient education and staff training, to repeat testing and obtain second‐technologist review when results are anomalous, and to establish a referral pathway to a regional or tertiary laboratory where molecular or confirmatory testing can be performed. Such measures would help distinguish true uropathogens from contaminants and improve the reliability of species identification where phenotypic methods are inconclusive.

### 4.3. Antimicrobial Susceptibility Patterns

Susceptibility testing revealed a clear pattern across drug classes: Aminoglycosides retained the highest activity against gram‐negative isolates (81.3% susceptible to both amikacin and gentamicin), whereas only 31.3% were susceptible to ampicillin and 62.5% were resistant to ceftriaxone. Susceptibility nonetheless varied considerably between organisms, and this pattern aligns with Ghanaian studies reporting high resistance to ampicillin, trimethoprim–sulfamethoxazole, chloramphenicol, ciprofloxacin, and ceftriaxone, whereas amikacin remained comparatively active [11, 12]. The reduced susceptibility observed to several *β*‐lactam antibiotics is consistent with the broader concern regarding *β*‐lactam resistance among uropathogens. However, the specific mechanisms underlying the resistance patterns observed in the present study were not investigated and therefore cannot be established from these data [22, 23]. Not all studies show identical patterns: In Ho, gentamicin susceptibility was 87% (comparable with 81.3% here), but resistance to amoxicillin–clavulanate, tetracycline, and fluoroquinolones was high [14]. Such divergence reflects differences in species composition, antimicrobial panels, prescribing practices, and laboratory methods. Organism‐level findings further illustrate heterogeneity: *E. coli* isolates were fully susceptible to amikacin, *K. pneumoniae* showed lower susceptibility, *P. mirabilis* was uniformly susceptible to aminoglycosides but resistant to ciprofloxacin, and *S. aureus* isolates showed high cotrimoxazole susceptibility, though denominators were small. Vancomycin results were excluded because disk diffusion is not definitive for *S. aureus*, and the single meropenem‐resistant *P. vulgaris* isolate cannot establish a broader carbapenem resistance trend [15]. Two *P. aeruginosa* isolates were excluded from susceptibility analysis due to apparent susceptibility to ampicillin and cotrimoxazole, inconsistent with intrinsic resistance mediated by the MexAB‐OprM efflux pump and chromosomal AmpC *β*‐lactamase [24]. This anomaly may reflect misidentification of nonfermentative gram‐negative rods or a rare permeability or efflux defect phenotype [25, 26]. Their exclusion reduced the number of interpretable isolates from 23 to 21 and should be considered when comparing with studies that included all recovered isolates. Taken together, these findings provide useful facility‐level surveillance data but are insufficient for empirical treatment selection given the small sample size and limited antimicrobial panel. They underscore the need for continued local susceptibility monitoring, periodic review of facility‐level antibiograms, and larger multicenter studies to determine whether these patterns are representative across Ghana.

### 4.4. Factors Associated With Culture‐Confirmed Significant Bacteriuria

Two behavioral factors were significantly associated with culture‐confirmed bacteriuria: tight underwear use (AOR = 6.56; 95% CI: 2.36–18.22; *p* < 0.001) and douching or genital deodorant use (AOR = 8.50; 95% CI: 2.83–25.54; *p* < 0.001). These estimates should be interpreted cautiously given only 23 positive events and wide CIs. The douching association is directionally consistent with pregnancy‐specific evidence from Northwest Ethiopia (AOR = 3.13; 95% CI: 1.10–8.92) [27], though other studies, including one in Addis Ababa, found no significant link [28]. Disruption of the *Lactobacillus*‐dominant vaginal microbiota is the most plausible mechanism, as repeated intravaginal product use can raise vaginal pH and reduce colonization resistance [29]; Ghanaian data similarly show intravaginal practices associated with low lactobacilli colonization in Accra [30]. Alternative explanations include unmeasured hygiene, sexual behavior, or socioeconomic factors. Evidence for tight clothing is less consistent. Although increased perineal heat and moisture could plausibly create an environment favorable to bacterial proliferation, this mechanism has not been directly established as a pathway to UTI. Moreover, available evidence has not demonstrated a consistent association between tight underwear or clothing and UTI. The large odds ratio observed may reflect sample characteristics, residual confounding, or sparse‐data effects. Maternal age and gestational age were not associated here, contrasting with findings from Ho, where first‐trimester pregnancy was linked to bacteriuria [14], and other studies identifying previous UTI, catheterization, parity, or socioeconomic factors [18]. The absence of associations in this study likely reflects limited power. Overall, these findings generate hypotheses for future research and support general genital‐health education in antenatal counseling, but tight underwear and douching should not be presented as proven causes. Larger prospective studies with systematic assessment of hygiene practices and confounders are needed to clarify these relationships.

### 4.5. Clinical and Public Health Implications

The findings have several implications for antenatal and laboratory practice. First, the detection of culture‐confirmed significant bacteriuria in 11.5% of participants demonstrates that bacterial growth meeting the study′s microbiological definition occurred among women attending antenatal care at the study facility. The finding is particularly relevant in settings where microbiological testing may not be routinely available, because clinical assessment alone cannot identify all women with ASB. Second, the susceptibility findings demonstrate that antimicrobial activity cannot be assumed to be uniform across bacterial species or healthcare settings. The relatively high susceptibility to amikacin and gentamicin, alongside substantial resistance to ampicillin and ceftriaxone, provides useful facility‐level surveillance information. However, these data are insufficient on their own to establish empirical treatment policy because of the small number of isolates, the single‐center design, and the exclusion of two *P. aeruginosa* isolates. Local susceptibility data should instead be interpreted alongside current national or facility treatment guidelines and updated periodically as resistance patterns change. The behavioral findings also suggest an area for antenatal health education, although the evidence should be communicated cautiously. General counseling on genital health and avoidance of potentially irritating or unnecessary genital practices may be appropriate, but the present study does not establish that tight underwear or douching causes bacteriuria. The findings are better viewed as identifying behaviors that warrant further investigation and may be considered within broader preventive health education. Finally, the differences between this study and other Ghanaian studies emphasize the need for multicenter surveillance. Data from district, regional, and tertiary facilities across different geographical areas would help determine whether the bacterial distribution and susceptibility patterns observed at this facility represent a local phenomenon or part of a wider pattern. Larger studies could also incorporate systematic assessment of urinary symptoms, previous antimicrobial exposure, sexual and reproductive history, and socioeconomic factors, together with molecular characterization of resistant organisms and their resistance mechanisms.

### 4.6. Limitations

This study has several limitations. Participants were recruited by convenience sampling from a single district‐level facility, which may have introduced selection bias and limits generalizability to the wider pregnant population in Ghana. The cross‐sectional design allowed assessment of associations but not of temporal or causal relationships. Because the primary outcome was microbiological rather than clinical, culture‐positive participants could not be classified as having ASB or symptomatic UTI. Only 23 participants had culture‐confirmed significant bacteriuria, resulting in small denominators for organism‐specific susceptibility estimates. The exclusion of two *P. aeruginosa* isolates because of unreliable susceptibility profiles and the recovery of only one *P. vulgaris* isolate further limited organism‐specific interpretation. The small number of events also restricted the multivariable model to two predictors; age and gestational age were not retained because of sparse cells, and the adjusted estimates, particularly those with wide CIs, should be interpreted as associations rather than definitive independent risk factors. Bacterial identification relied on conventional biochemical methods without molecular confirmation, and resistance mechanisms were not characterized. Vancomycin susceptibility was assessed by disk diffusion only and treated as preliminary because MIC confirmation was not possible. Nitrofurantoin and amoxicillin–clavulanic acid were not tested because disks were unavailable. Behavioral data were self‐reported and may have been affected by recall or social desirability bias. These limitations mean the findings should be interpreted as facility‐level evidence rather than estimates generalizable to all pregnant women in Ghana, and they support the need for larger multicenter studies with systematic clinical assessment and molecular characterization.

## 5. Conclusion

This study identified culture‐confirmed significant bacteriuria in 11.5% of pregnant women attending antenatal care at a district‐level facility in the Western Region of Ghana. *E. coli* was the predominant isolate, and susceptibility varied by species, with relatively high activity for amikacin and gentamicin among gram‐negative isolates, low susceptibility to ampicillin, and common resistance to ceftriaxone. Reported use of tight underwear and douching or genital deodorant use were associated with culture positivity. Because the study was single‐center, cross‐sectional, and based on a small number of isolates, these findings should be regarded as facility‐level surveillance data rather than definitive evidence for empirical treatment selection. They nevertheless support considering urine culture at the first antenatal visit where laboratory capacity permits, establishing or updating facility‐level antibiograms, reviewing empirical treatment guidance for bacteriuria in pregnancy in light of the high ampicillin and ceftriaxone resistance observed, and including counseling on avoiding douching and genital deodorant use in antenatal education, framed as prevention advice. Larger multicenter studies with molecular characterization of resistant isolates are needed to confirm these findings and determine whether they are representative of other settings in Ghana.

**Nomenclature**
ANC: antenatal care
ATCC: American Type Culture Collection
CLSI: Clinical and Laboratory Standards Institute
FTRCH: Father Thomas Rooney Catholic Hospital
UTI: urinary tract infection

## Author Contributions

Mina Ofosu conceptualized and designed the study, supervised the research project, provided resources, critically reviewed and revised the manuscript, and oversaw project administration. Vuur Edward Domar contributed to data collection, investigation, data curation, formal analysis, and preparation of the original manuscript draft. Patricia Sam Barko assisted with data collection, laboratory and field procedures, data validation and organization, and manuscript editing.

## Funding

No funding was received for this manuscript.

## Disclosure

All authors read and approved the final manuscript.

## Consent

The authors have nothing to report.

## Conflicts of Interest

The authors declare no conflicts of interest.

## Supporting information


**Supporting Information** Additional supporting information can be found online in the Supporting Information section. File S1: Study questionnaire used for data collection.

## Data Availability

Data are available on request from the authors.
