# Supplementary material for: Culture‐Confirmed Significant Bacteriuria and Antimicrobial Susceptibility Patterns Among Pregnant Women Attending Antenatal Care at a District Hospital in Ghana
Source: Biomed Res Int. 2026 Nov 14;2026:7197672. doi: 10.1155/bmri/7197672 (PMC13768936; doi:10.1155/bmri/7197672)
Supplement: Supplementary file 1 [file BMRI-2026-7197672-s001.pdf]

QUESTIONNAIRE

KUMASI TECHNICAL UNIVERSITY
DEPARTMENT OF LABORATORY TECHNOLOGY
FACULTY OF HEALTH SCIENCES

Dear Respondent,

I hope this message finds you well.

I am conducting a research study on "**Culture-Confirmed Significant Bacteriuria and Antimicrobial Susceptibility Patterns among Pregnant Women Attending Antenatal Care at a District Hospital in Ghana**." Your participation in this study is voluntary, and all responses will be kept strictly confidential.

The questionnaire is designed for academic purposes only, and your personal identity will not be disclosed. Completing this questionnaire should take approximately 10–15 minutes, and there are no right or wrong answers. Your honest opinions and experiences are highly valued. Thank you for taking the time to contribute to this research.

Consent

I have read the information above and agree to participate in this study.

☐ Yes, I agree to participate

☐ No, I do not agree to participate

Participant code: ______________ Date: ______________

SECTION 1: SOCIODEMOGRAPHIC INFORMATION

1. Age group (years)

a) 15–20
b) 21–26
c) 27–32
d) 33–38
e) Above 38

2. What is your occupation?

a) Farming

b) Teaching

c) Small-scale mining

d) Learning a trade

e) Trading/market seller

f) Unemployed/homemaker

g) Other (specify): _______________

3. What is your level of education?

a) No formal education

b) Basic

c) SHS

d) Tertiary

e) Non-formal education

SECTION 2: OBSTETRIC INFORMATION

4. Gestational age (weeks)

a) 1–12

b) 13–24

c) 25–36

d) Above 36

5. Parity (number of previous pregnancies carried beyond 20 weeks)

a) 0 (primigravida)

b) 1

c) 2–3

d) 4 or more

SECTION 3: CLINICAL INFORMATION

6. Have you experienced any urinary symptoms during this pregnancy?

a) Yes

b) No

7. If yes, what were the symptoms? (Tick all that apply)

a) Burning sensation

b) Frequent urination

c) Lower abdominal pain

d) Blood in urine

8. Have you ever been diagnosed with a urinary tract infection during your current pregnancy?

a) Yes

b) No

9. Have you received antibiotics for urinary symptoms or UTI treatment in the past two weeks?

a) Yes

b) No

10. Did your healthcare provider inform you about the importance of completing the full course of antibiotics prescribed for UTI?

a) Yes

b) No

11. Are you aware of any potential risks of untreated UTIs during pregnancy?

a) Yes

b) No

12. If yes, which of these? (Tick all that apply)

a) Pyelonephritis

b) Low birth weight

c) Preterm labour

d) Sepsis

SECTION 4: BEHAVIOURAL INFORMATION

13. Do you wear tight-fitting pants or underwear?

a) Yes

b) No

14. Do you have a history of douching or using vaginal deodorants?

a) Yes

b) No

15. If yes to Q14, what do you use? (Tick all that apply)

a) Vinegar and water solutions

b) Baking soda and water solutions

c) Antiseptics like povidone-iodine

d) Fragrance or scented products

Thank you for your participation.
